# Malignant JAK-signaling: at the interface of inflammation and malignant transformation

**DOI:** 10.1038/s41375-025-02569-8

**Published:** 2025-03-26

**Authors:** Florian Perner, Heike L. Pahl, Robert Zeiser, Florian H. Heidel

**Affiliations:** 1 Department of Hematology, Hemostasis, Oncology and Stem Cell Transplantation, Hannover Medical School (MHH), Hannover, Germany; 2 Department of Medicine I, Medical Center–University of Freiburg, Faculty of Medicine, University of Freiburg, Freiburg, Germany; 3 Leibniz-Institute on Aging, Fritz-Lipmann-Institute (FLI), Jena, Germany; 4 Cellular Therapy Center (CTC), Hannover Medical School (MHH), Hannover, Germany

**Keywords:** Myeloproliferative disease, Cell signalling, Myeloproliferative disease

## Abstract

The JAK pathway is central to mammalian cell communication, characterized by rapid responses, receptor versatility, and fine-tuned regulation. It involves Janus kinases (JAK1, JAK2, JAK3, TYK2), which are activated when natural ligands bind to receptors, leading to autophosphorylation and activation of STAT transcription factors [1, 2]. JAK-dependent signaling plays a pivotal role in coordinating cell communication networks across a broad spectrum of biological systems including development, immune responses, cell growth, and differentiation. JAKs are frequently mutated in the aging hematopoietic system [3, 4] and in hematopoietic cancers [5]. Thus, dysregulation of the pathway results in various diseases, including cancers and immune disorders. The binding of extracellular ligands to class I and II cytokine receptors initiates a critical signaling cascade through the activation of Janus kinases (JAKs). Upon ligand engagement, JAKs become activated and phosphorylate specific tyrosine residues on the receptor, creating docking sites for signal transducer and activator of transcription (STAT) proteins. Subsequent JAK-mediated phosphorylation of STATs enables their dimerization and nuclear translocation, where they function as transcription factors to modulate gene expression. Under physiological conditions, JAK-signaling is a tightly regulated mechanism that governs cellular responses to external cues, such as cytokines and growth factors, ensuring homeostasis and maintaining the functional integrity of tissues and organs. Highly defined regulation of JAK-signaling is essential for balancing cellular responses to inflammatory stimuli and growth signals, thus safeguarding tissue health. In contrast, dysregulated JAK-signaling results in chronic inflammation and unrestrained cellular proliferation associated with various diseases. Understanding the qualitative and quantitative differences at the interface of physiologic JAK-signaling and its aberrant activation in disease is crucial for the development of targeted therapies that precisely tune this pathway to target pathologic activation patterns while leaving homeostatic processes largely unaffected. Consequently, pharmaceutical research has targeted this pathway for drug development leading to the approval of several substances with different selectivity profiles towards individual JAKs. Yet, the precise impact of inhibitor selectivity and the complex interplay of different functional modules within normal and malignant cells remains incompletely understood. In this review, we summarize the current knowledge on JAK-signaling in health and disease and highlight recent advances and future directions in the field.

## Introduction

JAK-STAT (Janus Kinase-Signal Transducers and Activators of Transcription) signaling is crucial for transmitting signals from the cell surface to the nucleus, regulating immune responses, cell growth, and differentiation. Dysregulation, often caused by mutations, is linked to diseases like cancer and autoimmune disorders, making this pathway a key target for drug development. JAKs are activated upon ligand binding to cytokine receptors, leading to STAT protein phosphorylation and transcriptional activity. While normal JAK-STAT signaling maintains cellular homeostasis, persistent activation can drive diseases. Understanding these differences is vital for developing therapies that target diseased cells while preserving normal function. This review highlights current knowledge and recent advances in JAK-STAT research.

## The JAK-STAT signaling pathway

### Exploration of cell signaling

**Fig. 1:**
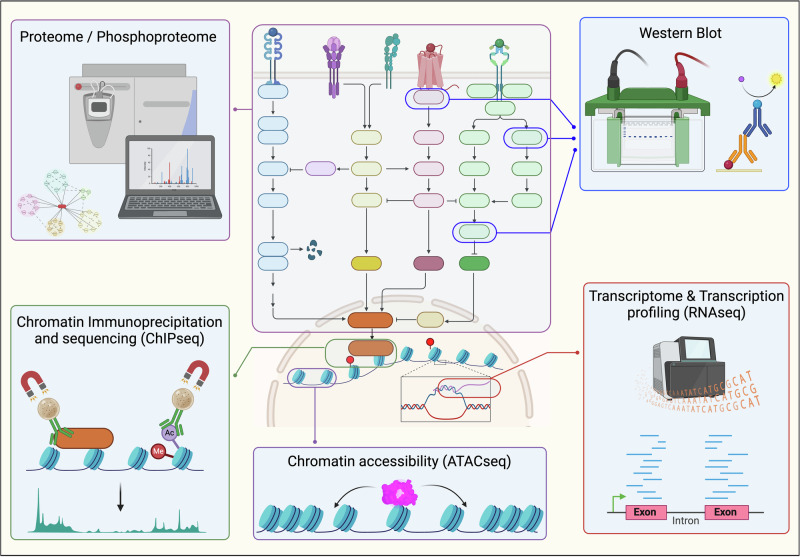
Overview of methods to study effectors and targets of cell signaling.

Experimental analysis of cell signaling has significantly evolved over the past few decades, transitioning from traditional methods like Western blot analyses to advanced techniques such as mass-spectrometry-based global analyses of post-translational modifications. Furthermore, recent advances in the accessibility of Next-Generation Sequencing (NGS) have enabled highly granular studies of downstream consequences of altered signaling cascades, such as genome-wide transcription factor binding patterns, post-translational modifications of histone tails, chromatin accessibility, and transcription. Together these technologies allow a holistic, multi-layered view of signal transduction processes and help to connect individual signaling events with a network of biochemical and functional processes (Fig. 1). In conjunction with evolving artificial intelligence tools, these advances may lead to the development of comprehensive mathematical models to accurately simulate complex networks of consequences from distinct signaling events in the near future.

Western blotting, a technique involving the separation of proteins by gel electrophoresis followed by the detection with specific antibodies, was the cornerstone of cell signaling research [6]. This method provided critical insights into protein expression levels and post-translational modifications, particularly phosphorylation events, but was limited by its semi-quantitative nature and the inability to provide comprehensive data on complex signaling networks. The advent of mass spectrometry-based global phosphoproteome analysis marked a transformative leap in the field [7–11].

Phosphoproteome analysis allows the high-throughput identification and quantification of thousands of phosphorylation sites across the proteome in a single experiment. The depth and breadth of data obtained through global phosphoproteomics enable researchers to construct detailed maps of signaling pathways and to understand dynamic changes in response to various stimuli or treatments on a holistic scale.

For the first time, this technique recently revealed the global phosphorylation landscape driven by malignant JAK2 signaling and exposed a critical mechanism of cancer cell persistence under JAK-inhibitor treatment [12, 13].

### Cytokine receptor signaling

**Fig. 2:**
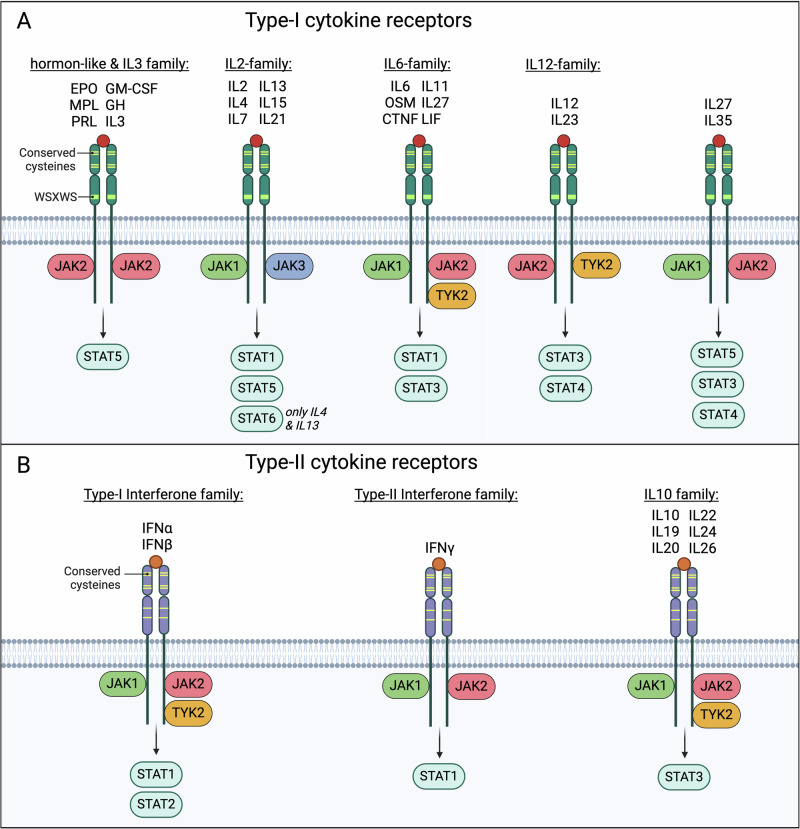
JAK-dependent cytokine receptor signaling. Schematic of type I (**A**) and type II (**B**) cytokine receptors and their association with JAKs and STATs. CTNF ciliary neurotrophic factor, EPO erythropoietin, GH growth hormone, GM-CSF granulocyte-macrophage colony-stimulating factor, IFN interferon, IL interleukin, LIF leukemia inhibitory factor, MPL thrombopoietin receptor, OSM oncostatin M, PRL prolactin.

Cytokine receptors constitute a pivotal component of the immune system, serving as crucial mediators of intercellular communication and regulation of immune responses. These receptors represent a diverse group of transmembrane proteins that play fundamental roles in various physiological processes, including immunity, hematopoiesis, and inflammation. Functionally, cytokine receptors are involved in transducing signals from extracellular cytokines to the intracellular environment, thereby orchestrating a wide array of cellular responses [14]. Type I and II cytokine receptors represent a highly conserved group of transmembrane proteins encompassing receptors for various cytokines such as interleukins (IL), interferons (IFN), erythropoietin (EPO), thrombopoietin (TPO), growth hormone (GH), leptin, and colony-stimulating factors [15, 16] (CSFs) (Fig. 2). Cytokine receptors lack intrinsic kinase activity but are associated with Janus kinases (JAKs) [17–22]. Upon ligand binding and subsequent activation of these receptors, JAKs undergo phosphorylation and in turn activate downstream signaling pathways. In recent years, cytokines and their respective receptors have been recognized as mediators of cellular homeostasis in different tissue microenvironments. Initially recognized for their function in the communication between immune cells, it subsequently became clear that cytokine-mediated signaling is a major language of communication between diverse cell types, including immune cells, neurons, epithelial cells, and connective tissue [14, 23]. Thus, dysregulation of cytokine secretion, cytokine receptor expression, and activation or downstream signaling have dramatic impacts on homeostasis and regeneration of hematopoietic and immune cell subsets in the central nervous system, vasculature, kidney, liver, lungs, and other organ systems [24–35]. JAK-dependent signal transduction is therefore of critical importance to orchestrate regeneration, repair, and defense in many organs and operates as a central mediator of cell communication [1].

### JAK-dependent signaling pathways

Janus kinases (JAKs) possess a carboxy-terminal catalytic or kinase domain, which is preceded by a pseudokinase or kinase-like domain. JAKs are constitutively bound to the intracellular domains of cytokine receptors through their amino-terminal FERM domain and SH2 domains [36]. Obtaining a detailed structure of a full-length JAK has been remarkably challenging. Recently, the structure of full-length JAK1, in complex with the intracellular domain of a cytokine receptor, has been solved [37]. This study revealed that the JAK1 domains form an extended structural unit, which facilitates dimerization of the cytokine receptor/JAK complex by close-packing of the pseudokinase domains from the monomeric receptor/kinase complex.

**Fig. 3:**
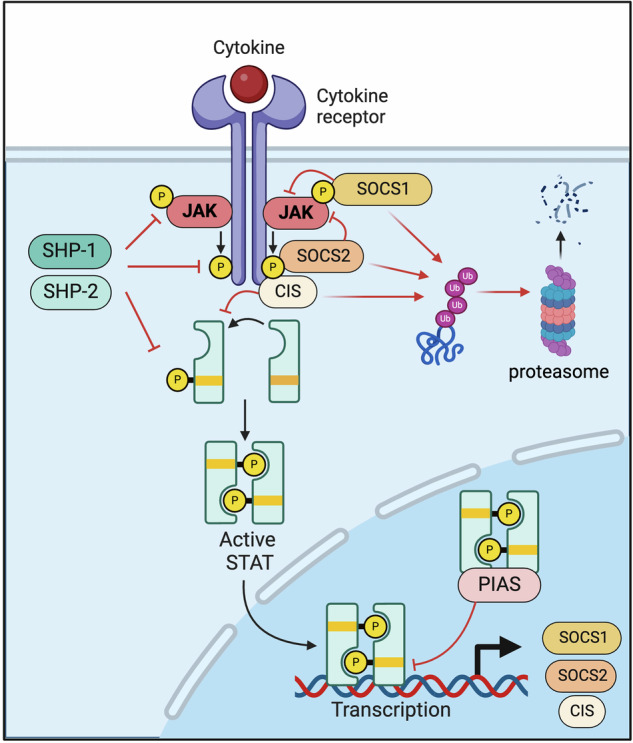
Regulation of the JAK-STAT signaling cascade. CIS cytokine inducible SH2 protein, JAK Janus kinase, PIAS Protein inhibitor of activated STAT, SHP Src homology region 2 domain-containing phosphatase, SOCS suppressor of cytokine signaling, STAT Signal Transducer and Activator of Transcription.

There are four known JAKs in humans–JAK1, JAK2, JAK3, and TYK2 [1, 36]. Each has specific yet overlapping roles in the downstream signaling of various cytokine receptors (Fig. 2). When a cytokine binds its receptor on the cell surface, it causes a conformational change that activates the associated JAKs. Once activated, JAKs phosphorylate specific tyrosine residues on the intracellular domain of the receptor, creating docking sites for signaling molecules [12] (Fig. 3). One of the key downstream effectors of JAK activation are STAT (Signal Transducers and Activators of Transcription) proteins. STATs constitute a family of transcription factors that play a critical role in conveying signals from the cell surface to the nucleus, resulting in the activation of gene expression [1, 36, 38]. They are key components of various cellular processes, particularly in response to cytokines and growth factors. Once phosphorylated, STAT proteins dimerize and translocate to the nucleus where they regulate the expression of specific genes (Fig. 3) [1, 36]. STAT proteins have an SH2 domain that allows them to bind to the phosphorylated tyrosine residues on the cytokine receptor. Upon receptor stimulation, JAK-mediated phosphorylation induces a conformational change in the STAT proteins, which allows dimerization, that is the pairing of two STAT molecules. Dimerization, which may be homologous or heterologous, involves the binding of a phosphorylated tyrosine in one STAT to the SH2 domain of another [36]. Dimerized STATs subsequently dissociate from the receptor, translocate to the nucleus [1, 39–41], and form an active transcription factor complex. Translocation is facilitated by their nuclear localization signal (NLS), which is recognized by nuclear transport receptors. In the nucleus, STAT dimers bind to specific DNA sequences, STAT binding motifs, in the promoter and enhancer regions of target genes, modulating their expression.

Janus kinases and their downstream effectors, STATs, are essential components of the signaling pathways triggered by various cytokines and growth factors and play a pivotal role in organ and organismic development, cell communication, immune responses, regeneration, blood cell formation, and tissue homeostasis.

### Negative regulators of JAK-signaling

Over the past decades, the mechanisms underlying JAK-STAT activation downstream of type I and type II cytokine receptors have been a major focus of research. Inhibitory mechanisms that counteract JAK-STAT activity and therefore balance responses to growth factors and inflammatory mediators have received less traction, but are critical for tissue homeostasis and preventing pathogenic pathway activation [42].

Protein-tyrosine-phosphatases (PTPs), specifically SHP-1 (PTPN6) and SHP-2 (PTPN11) were among the first negative regulators of JAK-signaling identified [43–45]. These molecules directly dephosphorylate JAKs and their associated receptors, and thereby counteract downstream signaling [46, 47]. Therapeutic modulation of PTPs is therefore evaluated in the context of autoimmune disorders and in tumor immunotherapy [48] but remains challenging in part due to the diverse functions of PTPs in dephosphorylating not only JAKs and their receptors but also a multitude of other intracellular signaling nodes.

In contrast, Suppressors of Cytokine Signaling (SOCS) family proteins are more selective negative regulators of JAK-STAT signals. SOCS proteins consist of 8 family members in humans (SOCS1-7 and CIS) and contain a central SH2 domain for the association with phosphorylated amino acid residues as well as a C-terminal SOCS domain that interacts with enlongin B and C as part of an E3 ligase complex [49–51]. SOCS-family proteins are typically expressed on a low level under steady-state conditions, but their transcription is induced as a negative feedback mechanism when JAK-dependent cytokine and growth factor receptors are activated [52, 53]. Among the members of the SOCS proteins, the molecular mechanisms of SOCS1, SOCS3, and CIS are best characterized. SOCS1 directly binds to phosphorylated JAKs via its SH2 domain and inhibits enzymatic activity by interfering with the JAK activation loop in a substrate-competitive manner [54]. In contrast, SOCS3 associates with phosphorylated residues of cytokine receptors, thereby binding in proximity to JAKs and inhibiting enzymatic activity via activation loop interactions similar to SOCS1. Furthermore, SOCS3 appears to compete with other substrates for binding to cytokine receptors therefore inhibiting downstream signaling by acting as a physical competitor for phospho-site accessibility [55, 56]. Likewise, CIS uses SHP2-domain dependent binding to phosphorylated receptor motifs to compete with STAT-proteins, preventing STAT recruitment and thereby inhibiting JAK-STAT signaling [47, 57]. In addition to these substrates- and binding-site competitive inhibitory mechanisms, members of the SOCS protein family promote E3-ligase recruitment via enlongin B and C, ubiquitination, and subsequent proteasomal degradation of JAK, STAT, and receptor proteins, thereby reducing the abundance of molecules available to promote JAK-STAT signaling [50, 58, 59].

Another family of molecules that negatively regulate JAK-STAT signals is “Protein Inhibitors of Activated STAT” (PIAS), consisting of PIAS1-4 [60]. PIAS proteins counteract STAT activity by binding to active STAT dimers and blocking DNA binding, thereby inhibiting transcription factor activity [61, 62]. Of note, PIAS activity is less selective for JAK-dependent signals compared to SOCS proteins, since they also intersect other transcription factor signals, including NFkB, and impact histone acetylation in a transcription factor-independent manner [60].

## Mutant JAK-signaling

Mutations, oncogenic fusions, and amplifications that affect JAK-signaling play a crucial role in malignant transformation by disrupting physiologic regulatory mechanisms and signaling networks, thereby leading to unregulated cell growth and survival. Affected genes include JAK1, JAK2, JAK3, and TYK2 as well as different STAT genes. Mutations in the JAK-STAT signaling pathway have been described in many different syndromes and clonal diseases and include acquired, somatic mutations as well as constitutive, germline aberrations.

### JAK2 mutations as drivers of myeloproliferative neoplasms

One of the most well-documented gain-of-function (GOF) mutations in the JAK-STAT pathway is JAK2-V617F, which is prevalent in myeloproliferative neoplasms (MPNs) including polycythemia vera, essential thrombocythemia, and primary myelofibrosis [63–66]. The V617F mutation, located in the JAK2 pseudokinase (PK) domain, leads to constitutive JAK2 activity, cytokine-independent, and uncontrolled proliferation [67], and resistance to apoptosis. The JAK2-V617F mutation constitutes the most common driver mutation in MPN [66, 68]. Similarly, JAK2 exon 12 mutations, including various insertions, deletions, and point mutations, although less common, result in persistent kinase activation and contribute to the development of polycythemia vera [69]. Of note, murine models showed that a single hematopoietic stem cell carrying a JAK2-V617F mutation is sufficient to cause MPN with erythrocytosis or thrombocytosis in vivo [70].

Bone-fide downstream effector pathways have been identified and studied, mostly by Western blotting in the context of JAK2-V617F, and functionally characterized using engineered mouse models. So far it is believed that STAT-proteins represent the primary downstream targets of JAKs. However, convincing time-resolution experiments on a global proteomic scale that would support this hypothesis are currently lacking. STAT5 as the primary STAT target downstream of critical hematopoietic growth factor receptors (including EPO-R, MPL, GM-CSF-R, and IL3-R) promotes the expression of genes involved in cell proliferation, survival, and differentiation [64, 71–73]. STAT3 and STAT1 can also be activated in JAK2-V617F mutant cells, contributing to the oncogenic signaling cascade downstream of other cytokine receptors [74, 75]. One large, unresolved question in the MPN field is the observation that the acquisition of the identical JAK2-V617F mutation can cause a variety of clinical phenotypes, ET, PV, or PMF. In this context, the specific pattern of STAT activation might influence the MPN phenotype as loss of STAT1 was shown to decrease megakaryopoiesis while favoring erythropoiesis in a mouse model of JAK2-V617F driven MPN [76].

PI3K/AKT signaling is another relevant pathway regulated by aberrant JAK signaling. PI3K/AKT controls cell survival and proliferation, with downstream activation of mTOR, GSK3, Chk1, and BAD, as well as NFkB and FOXO transcription factors [77, 78]. Likewise, MAPK/ERK signaling is activated by JAK2-V617F, causing subsequent activation of downstream effectors such as MNK1, MCL1, BIM, BAD, and YBX1, which prevent apoptosis induction and promote cell viability [12, 79–83]. Furthermore, RSK1 (Ribosomal S6 Kinase 1), a significant downstream effector in the MAPK-ERK signaling pathway, is activated in presence of a JAK2-V617F mutation [84]. Once ERK is activated by the upstream MAPK pathway, it phosphorylates RSK1 on several key serine and threonine residues. Activated RSK1 can phosphorylate various downstream effectors including the S6 kinase and other translation initiation factors. SOCS proteins, particularly SOCS3, are upregulated as a feedback mechanism but are often ineffective in inhibiting the hyperactivated JAK2-V617F signaling, leading to persistent pathway activation.

A recent study developed a conditionally inducible mouse model to investigate JAK2-V617F oncogene addiction [85]. The dual-recombinase system (Dre-rox/Cre-lox) allows precise control of sequential Jak2-V617F activation and inactivation, providing a robust model to studying oncogenic dependencies. Jak2-V617F deletion was more effective than pharmacologic JAK-inhibition in reducing mutant cell fraction, confirming that current inhibitors fail to achieve sufficient mutant kinase inhibition. Importantly, this study suggests that more selective and potent JAK2 inhibition may provide a larger therapeutic benefit.

### Other JAK mutations as drivers of blood cancer and immune system disorders

The first evidence of JAK gain-of-function mutations in hematological disorders, which preceded discovery of the JAK2-V617F mutation by 10 years, was provided by the Drosophila tumorous-lethal (Tum-l) mutation in the kinase-like domain of the hopscotch locus. Mutation of the hopscotch pseudokinase domain resulted in a malignant neoplasia of blood cells [86–88]. Resolution of a detailed structure of full-length JAK now provides insights into how PK mutations lead to cytokine-independent signaling (Table 1). Gain-of-function mutations are located within the core of the PK interdimer interface and enhance close-packing complementarity thereby facilitating ligand-independent activation [37]. JAK2 L611S, occasionally found in patients with AML, drives pathogenesis by sustained JAK2 activation [89]. Other JAK2 mutations associated with myeloid neoplasms and acute lymphoblastic leukemia (ALL) include R683G, R683S, and R683T, which drive malignant transformation by continuously activating downstream signaling cascades, resulting in increased proliferation of hematopoietic stem- and progenitor cells [90–93]. Activating mutations in JAK3, such as M511I, A572V, and A573V lead to aberrant signaling and transformation to acute megakaryoblastic leukemia or T-ALL [94–97] while inactivating mutations are associated with severe combined immunodeficiency (SCID) [98–100].

**Table 1:** Compilation of functionally validated and clinically relevant JAK-mutations.

Protein	Type	Alteration	Consequence	Disease/phenotype
JAK1	Mutation	p.R629_D630del [101]	Activating	- Chronic eosinophilic leukemia (CEL)
		A634D [102–104]	Activating	- Acute T-Lymphoblastic Leukemia (T-ALL) - Acute B-Lymphoblastic Leukemia (B-ALL) - Inborn errors of immunity (IEI) with severe allergic inflammation
		V658F [103]	Activating	- Acute T-Lymphoblastic Leukemia (T-ALL) - Acute B-Lymphoblastic Leukemia (B-ALL)
		R727H [102]	Activating	- Acute T-Lymphoblastic Leukemia (T-ALL) - Acute B-Lymphoblastic Leukemia (B-ALL)
		R879S/C/H [102]	Activating	- Acute T-Lymphoblastic Leukemia (T-ALL)
		multiple truncating and missense [105, 106]	Inactivating	- Immune-escape in diverse solid cancers
JAK2	Mutation	V617F [64, 66, 72, 85]	Activating	- Polycythemia vera (PV) - Essential thrombocythemia (ET) - Primary Myelofibrosis (PMF) - clonal hematopoiesis (CH)
		R683S/G/T [90–93]	Activating	- Acute T-Lymphoblastic Leukemia (T-ALL) - Familial thrombocytosis syndrome - Erythroid leukemia
		L611S [89]	Activating	- Acute Myeloid Leukemia (AML)
		R564L [315, 316]	Activating	- Acute Myeloid Leukemia (AML)
		I670V [316]	Activating	- Myelodysplastic neoplasms (MDS)
		Exon12 mutations between 536 and 547 (e.g., N542-E543del, E543-D544del or K539L) [69]	Activating	- Polycythemia vera (PV) - Familial Erythrocytosis
	Fusion	ETV6::JAK2 [113, 114]	Activating	- Myeloproliferative Neoplasms - Acute Myeloid Leukemia (AML) - Acute B-Lymphoblastic Leukemia (B-ALL)
		PCM1::JAK2 [117]	Activating	- Chronic eosinophilic leukemia (CEL)
		BCR::JAK2 [119]	Activating	- Myeloproliferative Neoplasia (MPN) - Acute Myeloid Leukemia (AML)
		PAX5::JAK2 [120]	Activating	- Acute B-Lymphoblastic Leukemia (B-ALL)
		TERF2::JAK2 [120]	Activating	- Acute B-Lymphoblastic Leukemia (B-ALL)
		GOLGA::JAK2 [121]	Activating	- Acute B-Lymphoblastic Leukemia (B-ALL)
		SEC31A::JAK2 [122]	Activating	- Hodgkin Lymphoma
		NFE2::JAK2 [116]	Activating	- Myelodysplastic neoplasms (MDS)
JAK3	Mutation	M511I [96]	Activating	- Acute T-Lymphoblastic Leukemia (T-ALL)
		A572V [95]	Activating	- Acute Megakaryoblastic Leukemia (AMKL)
		A573V [94, 96]	Activating	- Acute Megakaryoblastic Leukemia (AMKL) - Acute T-Lymphoblastic Leukemia (T-ALL)
		Q507P [317]	Activating	- Familial Chronic Lymphoproliferative Disorder of NK Cells
		V674A/F [318, 319]	Activating	- Acute T-Lymphoblastic Leukemia (T-ALL)
		R657Q [320]	Activating	- Acute T-Lymphoblastic Leukemia (T-ALL)
		multiple truncating and missense [100]	Inactivating	- SCID
	Fusion	JAK3::INSL3 [321]	Activating	- Cutaneous T-cell lymphoma (CTCL)
TYK2	Mutation	G36D [109]	Activating	- Acute T-Lymphoblastic Leukemia (T-ALL)
		S47N [109]	Activating	- Acute T-Lymphoblastic Leukemia (T-ALL)
		V731I [109]	Activating	- Acute T-Lymphoblastic Leukemia (T-ALL)
		E957D [109]	Activating	- Acute T-Lymphoblastic Leukemia (T-ALL)
		R1027H [109]	Activating	- Acute T-Lymphoblastic Leukemia (T-ALL)
		G761V [110]	Activating	- Acute T-Lymphoblastic Leukemia (T-ALL)
		P1104A [111]	Inactivating	- Breast-, Colon-, Stomach-cancer, AML
		P760L [111]	Activating	- Acute B-Lymphoblastic Leukemia (B-ALL)
		G671V [111]	Activating	- Acute T-Lymphoblastic Leukemia (T-ALL)
	Fusion	NPM1::TYK2 [111]	Activating	- Lymphoproliferative disorders (LDP)
		NFkB2::TYK2 [111]	Activating	- Anaplastic large-cell lymphoma (ALCL)
		ELAVL1::TYK2 [111]	Activating	- Acute Myeloid Leukemia (AML)
		PABPC4::TYK2 [111]	Activating	- Anaplastic large-cell lymphoma (ALCL)
		ETV6::TYK2 [111]	Activating	- Acute T-Lymphoblastic Leukemia (T-ALL)
		MYB::TYK2 [111]	Activating	- Acute B-Lymphoblastic Leukemia (B-ALL)

Mutations in JAK1, such as A634D, V658F, and p.R629_D630del, are rare drivers of hematologic malignancies, including chronic eosinophilic leukemia (CEL) [101], and inflammatory syndromes through sustained signaling activity [102–104]. In contrast, inactivating mutations in JAK1 are associated with solid cancers, likely due to immune evasion mechanisms [105–107], for example by altering the tumor microenvironment and abrogating INF-signaling, thereby causing resistance to PD-1 blockade [108]. Finally, activating TYK2 mutations have been implicated in certain leukemias, further highlighting the pervasive impact of dysregulated JAK signaling [109–111]. Similar to other JAKs, inactivating germline mutations in TYK2 are associated with immunodeficiency and cancer [111]. Specifically, P1104A, a loss-of-function variant of TYK2 is linked to increased infection susceptibility [112].

In addition to activating, inactivating, or truncating mutations, JAK and TYK2 can also drive malignant transformation as translocation partners in oncogenic fusion proteins. ETV6::JAK2 (TEL::JAK2) is found in various hematologic malignancies and results in constitutive kinase activity [113–116]. PCM1::JAK2, identified in chronic eosinophilic leukemia, leads to persistent JAK2 signaling [117, 118]. BCR::JAK2 is another rare fusion protein that is associated with MPN often accompanied by eosinophilia and AML [119]. In contrast, TERF2::JAK2, PAX5::JAK2, and GOLGA::JAK2-fusions are reported in ALL [120, 121]. SEC31A::JAK2 fusions are found in rare Hodgkin lymphoma cases and result in constitutive JAK2 activation [122]. Additionally, JAK2::NFE2 fusions were found in MDS [116]. TYK2 fusions such as MYB::TYK2, NPM1::TYK2, NFkB2::TYK2, and PABPC4::TYK2 are found in lymphomas and ALL leading to constitutive TYK2 signaling and contributing to malignant transformation [111, 123]. In summary, genetic alterations of Janus-kinases and subsequently altered downstream signaling facilitate malignant transformation by enabling cells to evade normal growth controls, resist programmed cell death, and sustain proliferation. Yet, the exact mechanisms by which aberrant JAK signaling establishes oncogenic networks and initiates malignant transformation are not sufficiently understood to date.

### Mutations in STAT-molecules

The functional role of STAT signaling molecules downstream of Janus-kinases has also been studied in the context of germline mutations causing a variety of syndromes: STAT1 and STAT2 loss-of-function mutations impair responses to interferons (IFNs), leading to susceptibility to viral and mycobacterial infections [124–126]. In contrast, gain-of-function (GOF) variants in STAT2 lead to excessive activation causing a lethal type-I interferonopathy [126–128]. STAT3 LOF mutations cause autosomal dominant HIES (Hyper-Immunoglobulin-E Syndrome), also known as Job's syndrome, characterized by elevated IgE levels, recurrent skin abscesses, and lung infections [129, 130]. STAT4 is essential for Th1 cell differentiation and IL-12 signaling. Consequently, LOF mutations in STAT4 result in impaired Th1 responses, leading to increased susceptibility to infections and a predisposition to autoimmune diseases due to inadequate cellular immunity [131–133]. STAT5 transcription factors include two closely related proteins, STAT5A and STAT5B. LOF mutations, particularly in STAT5B, cause immune dysregulation, growth hormone insensitivity, and recurrent infections [134, 135]. These mutations affect lymphocyte development and function, leading to growth impairment and a range of immunodeficiencies. The STAT5 N642H mutation is a potent driver of leukemogenesis, its expression is sufficient to induce leukemia in mice and it is frequently found in aggressive T-cell leukemia/lymphoma [136]. Replacement with a histidine causes a structural change in STAT5B, rendering it resistant to dephosphorylation, which promotes continuous activation and contributes to the aggressive nature of the leukemia [137]. STAT6 is pivotal for Th2 cell differentiation and IL-4/IL-13 signaling. LOF mutations in STAT6 result in defective Th2 responses, affecting the body’s ability to defend against helminth infections, while GOF mutations in STAT6 lead to an inherited allergic syndrome [138].

### JAK-inhibitors: targeted therapies for JAK-mutant cancers and inflammatory diseases

Because dysregulation of JAK-STAT signaling has been implicated in various disorders, JAK inhibitors have been developed as a therapeutic strategy and are currently approved in rheumatoid arthritis and certain subtypes of MPNs. In BCR:::ABL1-negative MPN, JAK inhibitors have shown significant clinical activity in reducing inflammation, thereby alleviating symptoms and reducing splenomegaly. Importantly, this effect is independent of the presence of the JAK2V-617F mutation, as the other driver mutations also result in increased JAK2 activity [139–143]. The JAK1/2 inhibitor ruxolitinib was therefore approved for the treatment of patients with myelofibrosis and polycythemia vera 10 years ago. To date, ruxolitinib is the most frequently prescribed JAK inhibitor in patients with MPN [143, 144], but advances in drug development have led to the introduction of highly selective inhibitors for individual JAKs allowing more precise targeting of specific disease mechanisms.

The far more JAK2-selective inhibitor fedratinib is now approved for the treatment of myelofibrosis and has demonstrated significant activity in a cohort of patients who had lost response to ruxolitinib treatment [145–148]. Interestingly, fedratinib-treated patients suffer from a higher degree of gastrointestinal (GI) -toxicity reflected by an increased incidence of nausea and diarrhea, but do not show systemic immunosuppression witnessed under unselective JAK1/2 inhibition. Following the SIMPLIFY (NCT01969838, NCT02101268) and MOMENTUM (NCT04173494) trials momelotinib, was recently approved. The inhibitor is unique in that it targets JAK1/JAK2 with added inhibition of ACVR1, the activin A receptor, thereby decreasing hepcidin transcription. So far momelotinib is the only JAK inhibitor that improves anemia, in some patients even alleviating transfusion dependency. Common side effects include mild GI symptoms and dizziness, but it lacks significant hematologic toxicity, a key advantage for those with existing cytopenias. Pacritinib is a selective JAK2/IRAK1 inhibitor approved for patients with MF and severe thrombocytopenia. The PERSIST trials (NCT01773187, NCT02055781) highlighted its efficacy in patients with low platelet counts, achieving spleen volume reduction and alleviating symptoms without the hematologic toxicity seen with other JAK inhibitors. Although pacritinib has a favorable hematologic safety profile, it may cause GI issues and, in rare cases, cardiovascular events, underscoring the importance of patient selection.

The rapid development of several selective and clinically active JAK inhibitors in the last decade underscores the pivotal role that JAK-STAT signaling plays in MPN pathophysiology (Table 2). Very recent data from the MAJIC-PV (ISRCTN61925716) trial showed for the first time that molecular remissions are attainable with JAK-inhibition by ruxolitinib and that patients who achieve a molecular remission have enhanced event free survival. A comparison between different clinically available JAK inhibitors regarding their selectivity, activity, and safety profile is provided in Table 3 [149].

**Table 2:** Clinically available JAK-inhibitors (FDA- or EMA-approved or in advanced clinical trials).

JAK-inhibitor	Selectivity	Clinical stage	Major areas of application
Ruxolitinib	JAK1/JAK2	FDA approved EMA approved	Approved for: Myelofibrosis, Polycythemia vera, acute and chronic GVHD Clinical trials: Alopezia areata, Atopic dermatitis, Psoriasis, T-ALL, Essential thrombocythemia, COVID-19, Hemophagocytic Lymphohistiocytosis, Pancreatic Cancer, Vitiligo
Momelotinib	JAK1 / JAK2	FDA approved EMA approved	Approved for: Myelofibrosis Clinical trials: Polycythemia vera, Essential thrombocythemia, Metastatic Pancreatic Ductal Adenocarcinoma,
Fedratinib	JAK2	FDA approved EMA approved	Approved for: Myelofibrosis
Pacritinib	JAK2/FLT3	FDA approved	Approved for: Myelofibrosis
Tofacitinib	JAK1/JAK2/JAK3	FDA approved EMA approved	Approved for: Rheumatoid arthritis, Juvenile idiopathic arthritis, Ulcerative colitis, Psoriatic arthritis Clinical trials: Alopecia areata, Psoriasis, Ankylosing spondylitis, Takayasu arteritis
Baricitinib	JAK1/JAK2	FDA approved EMA approved	Approved for: Rheumatoid arthritis, Atopic dermatitis, COVID-19 Clinical trials: Juvenile idiopathic arthritis, Alopecia areata, Systemic lupus erythematosus
Peficitinib	JAK3 (>JAK1/JAK2/TYK2)	FDA approved EMA approved	Approved for: Rheumatoid arthritis, Clinical trials:
Upadacitinib	JAK1 (>JAK2)	FDA approved EMA approved	Approved for: Rheumatoid arthritis, Ankylosing spondylitis, Psoriatic arthritis Clinical trials: Atopic dermatitis, Axial spondyloarthritis, Ulcerative colitis, Crohn’s disease, Takayasu arteritis, Giant cell arteritis
Filgotinib	JAK1 ( > JAK2)	FDA approved EMA approved	Approved for: Rheumatoid arthritis, Clinical trials: Psoriatic arthritis, Ulcerative colitis, Crohn’s disease
Deucravacitinib	TYK2	phase 3	Clinical trials: Psoriasis
Ritlecitinib	JAK3	phase 3	Clinical trials: Alopecia areata
Izencitinib	JAK1/JAK2/JAK3/TYK2	phase 3	Clinical trials: Crohn’s disease

*EMA* European medical agency, *FDA* US food and drug administration.

**Table 3:** Comparison of approved JAK inhibitors in MPN.

Characteristic	Ruxolitinib	Fedratinib	Pacritinib	Momelotinib
Kinase targets	JAK1, JAK2, TYK2	JAK2, FLT3	JAK2, FLT3, IRAK1	JAK1, JAK2, ACVR1
Clinical trials	COMFORT I + II (NCT00952289, NCT00934544)	JAKARTA, JAKARTA-2 (NCT01437787, NCT01523171)	PERSIST-1, PERSIST-2, PAC203 (NCT01773187, NCT02055781, NCT04884191)	SIMPLIFY-1, SIMPLIFY-2, MOMENTUM (NCT01969838, NCT02101268, NCT04173494)
Spleen response(% of patients with > 35% reduction at 24 weeks)	32–41.9%	36–47%	19%	26.5%
Symptom response (% of patients with > 50% reduction at 24 weeks)	45.9%	36%	41%	28.4%
Anemia (grade 3/4)	45.2%	43%	17%	13.6%
Thrombocytopenia (grade 3/4)	12.9%	17%	12%	18.7%
Other typical adverse events	Muscle cramps, Herpes reactivations, rare events related to immunosuppression (opportunistic infections, Merkel cell carcinoma)	Nausea, vomiting, diarrhea, Wernicke's encephalopathy, and other conditions related to thiamin deficiency (rare)	GI symptoms, cardiac events related to QTc prolongation, or heart failure (rare)	Polyneuropathy
Dose	5–25 mg BID	400 mg QD	400 mg QD	200 mg QD

*BID* "bis in die" (latin: twice a day), *QD* "quaque die" (latin once a day).

Pathogenic JAK1/2 activation was also shown to drive inflammation during graft-versus-host disease (GVHD) in mice and patients [150]. Two randomized phase III trials showed that ruxolitinib was superior to the best available therapy for corticosteroid refractory acute and chronic GVHD [151, 152], respectively, which led to FDA and EMA approval for this indication.

JAK-inhibitors with broad activities have also been approved in diverse inflammatory diseases [153] including rheumatoid arthritis, psoriatic arthritis, atopic dermatitis, ulcerative colitis, and ankylosing spondylitis, with tofacitinib being the most commonly used compound. In rheumatologic diseases, the JAK1-selective inhibitors upadacitinib and filgotinib show similar immunosuppressive potential, while diminishing hematologic toxicity caused by JAK2-inhibition, although the biochemical selectivity for JAK1 over JAK2 is just about 2-fold [154, 155]. The highly JAK3-selective inhibitor ritlecitinib [156] and the TYK2-selective inhibitor deucravacitinib [157] are also evaluated in advanced clinical trials to date. Table 2 provides an overview of clinically available JAK inhibitors, their selectivity profile, and their area of application.

## Molecular targets of malignant JAK-signaling

### Transcriptional regulation of cell growth and inflammation

The inflammatory phenotype observed in MPN patients is a major aspect of JAK2-induced pathophysiology and contributes to morbidity and mortality [158]. High levels of pro-inflammatory cytokines were detected in murine MPN models as well as in primary patient samples [159–162]. Of note, high levels of cytokine expression are prognostically relevant indicating adverse outcomes in patients with MPN [161]. Cytokine and chemokine expression is highly regulated on the transcriptional level by a network of transcription factors, including STATs [163]. STATs bind target sites that are often characterized by groupings of conserved motifs bearing core sequences of TTCT/CNA/GGAA [164]. Across mammalian genomes, approximately one million of these sequences can be found of which roughly 10% are bound by STAT molecules [165]. The presence of multiple binding sites in special proximity facilitates the engagement of additional STAT molecules, thereby fostering accelerated transcriptional activity downstream of JAK-STAT activation [164–166].

STAT-regulated expression of growth- and inflammatory factors is an evolutionarily conserved cellular response mechanism following stimulation of JAK-dependent receptors [39, 40, 167], yet a specific set of key transcriptional targets have been characterized in the presence of the JAK2-V617F mutation [168, 169]. For example, tumor necrosis factor-alpha (TNFα) is highly expressed by *JAK2*-mutated MPN cells, and its levels correlate with disease burden in primary patient cells [170]. Inactivation of TNFα in murine models of MPN or in primary patient cells resulted in reduced disease development in vivo and abrogated clonal growth ex vivo, indicating its functional importance as an effector of JAK2-V617F signaling. Likewise, JAK2-V617F has been shown to be associated with increased expression of IL6 and loss-of-function polymorphisms in the IL6-receptor protection from MPN [74, 171, 172]. Furthermore, the JAK2-V617F mutation is associated with increased expression of the chemokine CXCL10 [160].

Recently, phospho-proteomic analysis to evaluate the global signaling landscape downstream of mutant JAK2-kinase has been reported [12]. This information offers a first objective, global perspective on pertinent downstream effectors of mutant JAK2. Of note, several transcription factors aside from STATs have been identified as bona-fide targets of JAK2-V617F signaling, including MYC, TP53, and JUNB [12]. This finding indicates a complex regulation of transcription factors and transcriptional networks by oncogenic JAK2-signaling, but the precise trajectories and functional consequences of JAK-dependent differential phosphorylation of non-STAT transcription factors remain elusive so far.

### Spliceosome and pre-mRNA-processing

A comprehensive analysis of JAK2-regulated proteins using mass-spectrometry-based proteomics and phospho-proteomics revealed a global map of bona fide JAK2 targets, including expected alterations in the phosphorylation status of cyclin-dependent kinases (CDKs), glycogen synthase kinase-3 (GSK3), proto-oncogene serine/threonine-protein kinase (PIM), and STAT5 [12]. Of note, "*mRNA splicing and processing*" was identified by gene ontology analysis as one of the most highly enriched biological processes in JAK2-mutated cells. In fact, several splicing factors and proteins involved in mRNA processing were differentially phosphorylated in JAK2-V617F mutant cells. This is a finding of great interest because mutations affecting the spliceosome machinery have been found in advanced phases of MPN [173], and certain mutations, like U2AF1, or Serine and arginine-rich splicing Factor 2 (SRSF2), are included in advanced scoring systems, as they are considered prognostically significant [174].

Landmark studies have demonstrated how therapeutic targeting of myeloid malignancies containing spliceosome factor mutations might be facilitated by modulating splicing catalysis [175]. They demonstrated that SRSF2 mutations, which are usually heterozygous in human myelodysplastic neoplasms (MDS) and AML, depend on the wildtype allele for survival in mouse models of both diseases. It was discovered that SRSF2-mutant cells appear more sensitive than non-mutated cells when the spliceosome machinery was pharmacologically perturbed. Results identified by global phosphoproteome analysis now shed additional insight on the matter, emphasizing that the spliceosome machinery is hijacked by an oncogenic kinase through post-translational alteration as opposed to the splicing factor itself being mutated. This recent report [12] highlighted the necessity of tightly controlled mRNA splicing of the ERK-signaling component Mitogen-activated Protein Kinase Interacting Serine/Threonine Kinase 1 (MKNK1) via phosphorylation of splicing factor Y-box Protein 1 (YBX1) by mutant JAK2. In turn, MKNK1 induces an ERK-phosphorylation rebound despite JAK inhibition and is necessary for the upkeep of the JAK2-mutant cells throughout JAK-inhibitor treatment. Targeting of the splicing factor YBX1 or MKNK1/ERK-signaling resolved the persistence of JAK2-V617F mutant cells under JAK-inhibitor therapy while leaving cellular fitness in the absence of the drug largely unaffected, demonstrating that JAK-dependent control of oncogenic mRNA splicing is a critical function specifically for the phenomenon of MPN persistence under kinase inhibitor exposure.

### Modification of the chromatin landscape

**Fig. 4:**
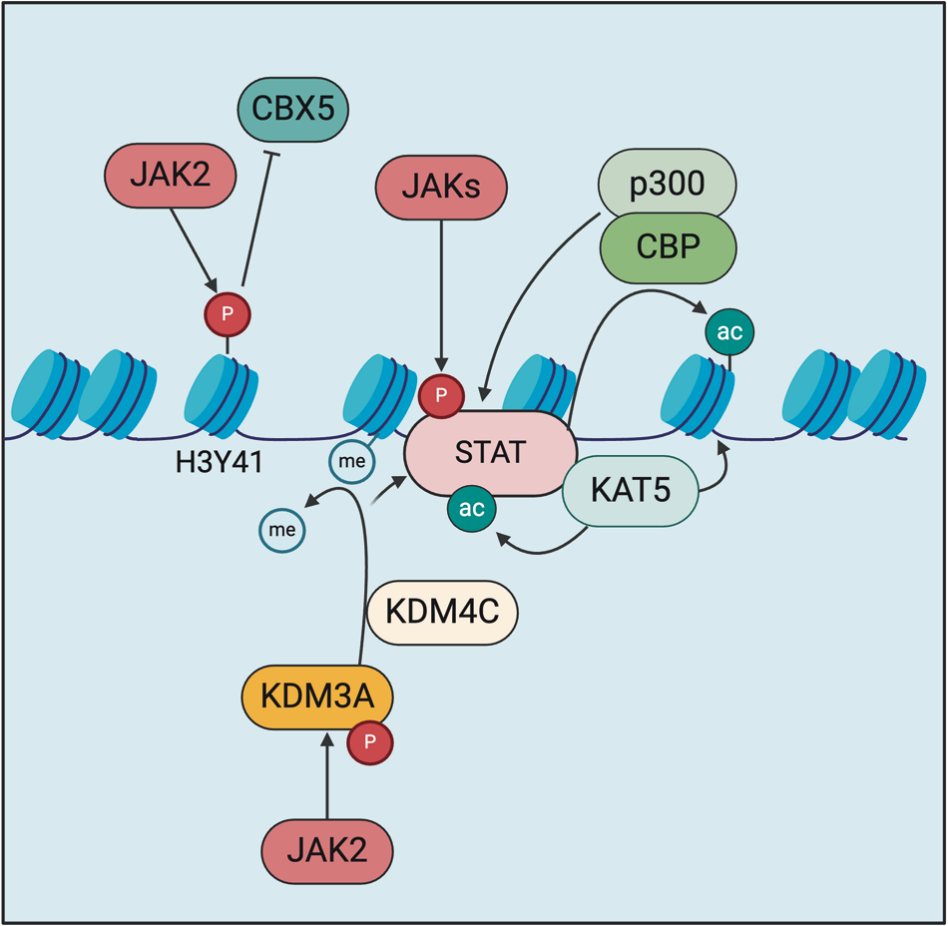
JAK-signaling into chromatin. CBP CREB-binding protein, CBX Chromobox protein, JAK Janus kinase, KDM lysine demethylase, KAT lysine acetyltransferase, p300 Histone acetyltransferase p300, STAT Signal Transducer and Activator of Transcription.

Epigenetic modifications, including DNA methylation, posttranslational modifications of histone tails, and chromatin remodeling are critical determinants of stem cell functions, cell fate decisions, and malignant transformation [176, 177]. Aberrant JAK2 signaling impacts the epigenetic landscape directly as well as by secondary mechanisms (Fig. 4). Several such mechanisms have been described in recent years, still JAK2’s complex role as a modulator of epigenetic cell states has not received much attention in the field so far.

Of note, JAK2 is present in the nucleus and is itself a writer of histone phosphorylation [178, 179]. JAK2 is capable of phosphorylating histone H3 at tyrosine 41 (H3Y41) both in vitro and in vivo [180]. Phosphorylation of H3Y41 restricts binding of HP1a (CBX5) to chromatin leading to loss of HP1a at sites where H3Y41 is present. Pharmacologic JAK inhibition resulted in a marked reduction in H3Y41 phosphorylation indicating a direct and non-redundant dependency on JAK enzymatic activity [180]. JAK2 activity thereby aids in stabilizing a permissive chromatin state since HP1a promotes the stability of heterochromatin [181]. Exclusion of HP1a by JAK-dependent H3Y41 phosphorylation consequently promotes euchromatin features at the involved sites, which include promotors and gene-body regions of active genes as well as cis-regulatory elements [182]. For example, expression of the transcription factor NFE2, which is overexpressed in the majority of MPN patients, is regulated through the epigenetic JAK2 pathway by phosphorylation of H3Y41 [183]. JAK2-V617F-dependent signaling into chromatin has further been demonstrated to promote the expression of the Homeobox protein NANOG as a critical mediator of cytokine independence [184]. JAK2-dependent phosphorylation has also been described as a critical mechanism to regulate the activity of other chromatin modifiers. For example, nuclear JAK2 directly phosphorylates and activates the Histone demethylase KDM3A which in turn modifies H3K9-methylation at STAT3 binding sites to assure permissive chromatin at the respective enhancer regions [185]. It is to be anticipated that more nuclear proteins are phosphorylated by JAK2, but too little data is available to estimate the full spectrum of directly JAK-dependent epigenetic writers, readers, and erasers to date.

The histone demethylases KDM4C (JMJD2C) and KDM3C (JMJD1C*)* were recently identified as signaling effectors of mutated JAK2 and target genes of the transcription factor NFE2, which is overexpressed in myeloproliferative neoplasms [183, 186, 187]. Depleting KDM3C expression significantly reduced cytokine-independent growth of an MPN cell line. KDM4C is a selective genetic dependency in JAK2-mutated cells, even under JAK inhibitor treatment. Genetic inactivation of KDM4C led to reduced proliferation and increased cellular senescence in JAK2-mutated cells, both in vitro and in vivo.

Another layer of chromatin landscape regulation secondary to JAK-activation is histone acetylation of regulatory elements mediated by activated STATs. It had long been recognized that in addition to motif-dependent DNA binding of activated dimers, STAT proteins activate chromatin by recruitment of the histone acetyltransferases p300 and CBP via their transactivation domains [188–190]. Similarly, STAT-dimers have been demonstrated to associate with the histone acetyltransferase TIP60 (KAT5) [191]. Of note, STAT protein activity itself is also regulated by acetylation, therefore, the association with acetyltransferases impacts both the activation state of chromatin around STAT binding sites and the catalytic activity of STATs [192–194]. JAK-STAT signaling in MPN therefore establishes aberrant histone acetylation, particularly at cis-regulatory elements regulated by inflammatory pathways, including TNFa-NfkB. Acetyl-reader proteins like BRD4 associate with these sites and are critical for the activation of inflammatory gene expression programs and concomitant inhibition of JAK-signaling and bromodomain function silences aberrant cytokine production and decreases the fitness of MPN cells [195–197]. Bromodomain (BET) inhibitors against BRD4, such as pelabresib, target both oncogenic JAK/STAT signaling and inflammatory NF-κB pathways in myeloproliferative neoplasms (MPNs). Preclinical studies demonstrated that BET inhibition reduces NF-κB-driven inflammation, cytokine production, and bone marrow fibrosis, with enhanced therapeutic effects when combined with JAK inhibitors. The Phase 2 MANIFEST (NCT02158858) trial showed that pelabresib, both alone and in combination with ruxolitinib, significantly improved spleen volume, symptom burden, and bone marrow fibrosis while lowering inflammatory cytokines and mutant allele fractions [198]. The Phase 3 MANIFEST-2 (NCT04603495) study further confirmed that pelabresib-ruxolitinib led to superior spleen response rates (65.9% vs. 35.2%) and meaningful improvements in bone marrow morphology compared to ruxolitinib alone [199]. Additionally, the combination therapy improved hemoglobin levels and reduced transfusion dependency, with manageable adverse effects like anemia and thrombocytopenia. These findings position pelabresib as a potentially transformative therapy for MF, addressing inflammation, fibrosis, and disease persistence beyond JAK inhibition alone. Of note, oncogenic mutations in calreticulin (CALR), which aberrantly activate TPO-signaling are the second most frequent driver of MPN after JAK2-V617F are particularly associated with sensitivity to BET- and HDAC-inhibitors [200].

The histone demethylase LSD1 (KDM1A) has been shown to be a genotype-selective, targetable dependency in MPN [201, 202]. LSD1 inhibitors, such as bomedemstat, selectively impair malignant clones, reduce inflammatory cytokines, and modify disease by lowering mutant allele burden and bone marrow fibrosis. Clinical trials have shown that bomedemstat normalizes platelet counts in essential thrombocythemia (ET) and reduces spleen volume, fibrosis, and symptoms in myelofibrosis (MF) [201, 203–206]. Ongoing clinical trials are further investigating LSD1 inhibitors, including their combination with JAK inhibitors, to optimize treatment outcomes for high-risk MPN patients.

In addition to its impact on chromatin, JAK2-signaling and the presence of a JAK2-V617F mutation also influence DNA methylation by phosphorylation of the DNA hydroxymethylase TET2, leading to a global decrease in DNA methylation [207]. In summary, JAK2-signaling influences epigenetic regulation in multiple ways but it is likely that many other epigenetic mechanisms by which the oncogene influences cell state and cell fate have not been discovered to date.

### Metabolism and energy consumption

Growth factor and cytokine signals transmitted by JAK/STAT signaling, including central inflammatory mediators like interferon-gamma and IL6, are important mediators of cellular activation, stress responses, and therefore energy consumption. Consequently, JAK-STAT signaling is tightly intertwined with the metabolic cell state and mitochondrial functions [25, 208–210]. Interestingly, common single nucleotide polymorphisms in the JAK2 gene are associated a with reduced risk for metabolic syndrome and related disorders [211]. Furthermore, JAK2 has been demonstrated to be critical for thermogenesis, beta cell function, adipose tissue function, and liver metabolism [212–215]. In macrophages, JAK2 is critical for high-fat diet-induced systemic inflammation [216] and has been demonstrated to be a critical mediator of beige adipose tissue formation and therefore thermogenesis [217]. Mutant JAK2-signaling led to changes in cell metabolism including hypoglycemia and adipose tissue atrophy in mouse models of MPN [218]. This phenotype correlated with hyperproliferative erythropoiesis in mice due to a combination of elevated glycolysis and increased oxidative phosphorylation. Here, nutrient supply through a high-fat diet improved survival of animals with JAK2-mutated hematopoiesis, while a high-glucose diet augmented the myeloproliferative phenotype [218]. This work links metabolic phenotypes that can also be observed in patients directly to cell-intrinsic functions of the JAK2-V617F oncogene rather than considering these phenomena solely as a secondary consequence of the disease. Interestingly, one challenging side effect of ruxolitinib treatment is undesired weight gain. Molecularly, this is explained by interference with leptin signaling. Leptin is released by adipocytes to signal satiety. The leptin receptor, (Lep-R, Ob-R) signals through JAK/STAT, and this pathway is likewise attenuated by JAK-inhibition [208]. Furthermore, JAK2-V617F signaling in macrophages drives a hyperinflammatory pathogenic phenotype, thereby promoting vascular inflammation and damage [219]. A recent study linked hyperreactive platelets and proinflammatory cytokine secretion in MPN patients to a specific metabolic state with increased ATP production and reduced α-ketoglutarate (α-KG) levels. Interestingly, α-KG supplementation in an MPN mouse model reduced pathogenic platelet activation, limited proinflammatory cytokine release from monocytes, and led to a decrease in spleen size [220].

### Cell adhesion and thrombosis

Cytokine-mediated immune cell activation triggers “inside-out” signaling of integrins increasing their affinity to cadherin and selectin ligands [221]. Activation of JAKs in immune cells leads to phosphorylation of Rap1 which in turn binds to and activates kindlins to cause a switch of integrin molecules towards an active conformation [222]. JAK2 deficiency in platelets severely impacts integrin activation and causes a coagulation defect leading to bleeding diathesis [223]. Interestingly, balanced activation of integrins is not only critical for coagulation and diapedesis of immune cells but also for hematopoiesis since integrin-ligand interactions anchor stem cells to the niche. Consequently, kindlin deficiency leads to impaired erythropoiesis in a murine model system [224]. It has been demonstrated that stimulation of this pathway by oncogenic kinase signaling is required for homing of leukemic stem cells to their niche in the bone marrow [225]. The JAK2-V617F mutation similarly stimulates this signaling axis leading to the activation of β1-integrin causing augmented adhesion of JAK2-mutant primary patient-derived granulocytes or a myeloid progenitor cell line in vitro [226]. Primary murine cells harboring a JAK2-V617F mutation show an increased affinity to both VCAM1 and ICAM1 ligands [227]. Increased integrin activation was attributed to the activation of Rap1 stimulating inside-out signaling. Importantly, it was demonstrated that this increased integrin activation is causative for pathogenic thrombosis. Antibody-mediated blockade of VLA-4 or β2-integrin might therefore prevent JAK2-V617F-mediated coagulopathy [227].

### Apoptosis and survival

JAK-signaling prevents apoptosis and promotes cellular survival through several key mechanisms. Activation of the JAK-STAT pathway upregulates the expression of anti-apoptotic genes, such as BCL-2, BCL-XL, and MCL-1 [228–230]. These proteins inhibit the intrinsic (mitochondrial) pathway of apoptosis by preventing the release of cytochrome c required for caspase activation and apoptosis induction [231]. Furthermore, JAK-STAT signaling can downregulate the expression of pro-apoptotic factors such as BAX and BAD [232, 233], which promote apoptosis by antagonizing the function of BCL-2 family proteins [231]. Activated STAT3 and STAT5 proteins directly regulate genes that promote proliferation and cell cycle progression and oppose cell death. Therefore, STATs directly control pro-survival programs in different cellular contexts [165, 234, 235]. In breast cancer, it has been demonstrated, that induction of interferon (IFN) signaling protects cancer cells from DNA damage-induced cell death. The expression of a 7-gene “IFN-related DNA damage resistance gene signature” (IRDS) predicted the efficacy of adjuvant chemotherapy and radiation [236]. In JAK2-V617F mutant MPN, the MAPK pathway promotes survival under JAK-inhibitor treatment in a JAK2-V617F-dependent manner through activation of ERK. In preclinical model systems, only combined inhibition of JAK-STAT and MAPK signaling allowed a reduction in clonal burden and disease eradication in some cases [12, 82]. Utilizing single-cell RNA sequencing on patient-derived stem and progenitor cells, aberrant DUSP6 expression was identified as a key driver of disease transformation and cell survival in MPN [84]. Ectopic expression of DUSP6 in patient-derived xenograft (PDX) models conferred resistance to JAK inhibitors and exacerbated disease severity. Conversely, DUSP6 inhibition effectively suppressed disease progression in Jak2-V617F driven disease. Inactivation of the DUSP6-RSK1 axis suppressed JAK2-mediated S6 signaling but also significantly impacted cell viability, induced apoptosis, and cell cycle arrest in JAK2-mutant cells. Furthermore, mechanisms by which JAK2 inhibition induces apoptosis in cells with constitutively active JAK2 mutations, particularly focusing on the role of the pro-apoptotic protein Bim, have been identified [237]. This study demonstrated that JAK2 inhibition leads to the upregulation of unphosphorylated Bim, which is crucial for the induction of apoptosis in JAK2-mutant hematopoietic cells. Treatment with JAK inhibitor I resulted in the upregulation of BIM in its active, unphosphorylated form in JAK2-mutant cell lines. This process was accompanied by the downregulation of the anti-apoptotic protein BCL-XL. The inhibition of ERK1/2 signaling was found to be a significant mediator of BIM activation. ERK inhibition prevented BIM degradation, leading to its accumulation and pro-apoptotic activity. The BH3 mimetic ABT-737, which mimics the action of BH3-only proteins like BIM, was able to enhance apoptosis when combined with JAK2 inhibition [237]. Moreover, JAK2-V617F promotes the accumulation of reactive oxygen species (ROS) by dysregulation of PI3K signaling. In addition, PI3K pathway activation decreases nuclear localization FOXO3A, resulting in reduced transcriptional activity. Inhibition of PI3K restores nuclear FOXO3A localization, increases catalase levels, reduces ROS, and sensitizes cells to DNA-damage-induced apoptosis [238].

Studies on the role of NfkB signaling provided the first evidence that Jak2 and Ikk2 cooperate to ensure proper hematopoietic cell function. Jak2 signaling supports the proliferation and differentiation of hematopoietic stem and progenitor cells, while Ikk2-mediated NF-κB activation promotes survival and anti-apoptotic responses [239]. Jak2 and Ikk2 can also exert opposing effects. Overactivation of Jak2 can lead to excessive cell proliferation, while Ikk2 can mitigate this by enhancing apoptotic responses through NF-κB signaling, maintaining a balance in hematopoietic cell populations. In the context of constitutively activated Jak2, conditional Ikk2 deletion led to increased apoptosis and reduced cell survival [239].

### Bone marrow niche and microenvironment

**Fig. 5:**
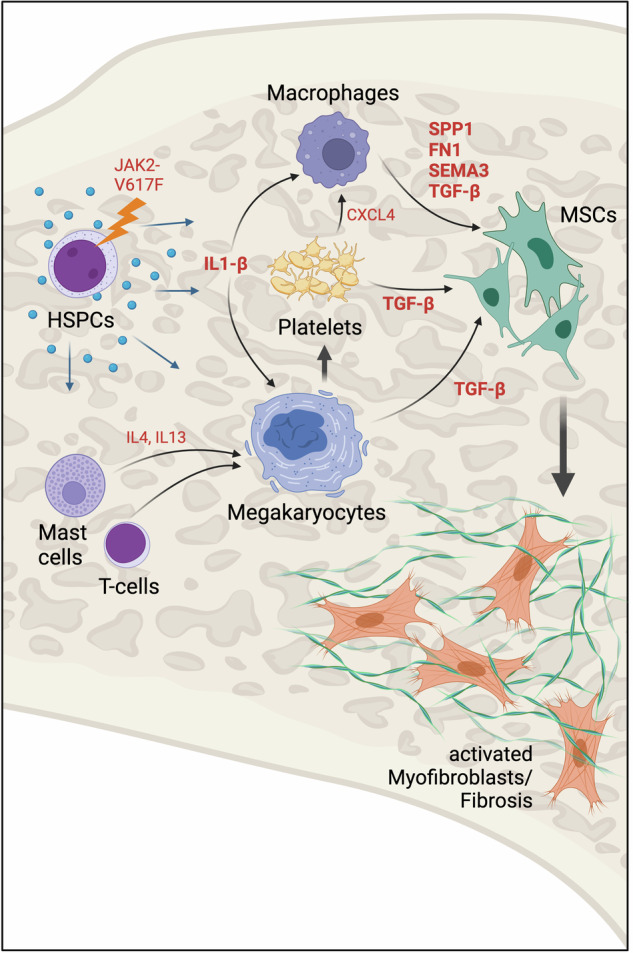
Aberrant activation of the bone marrow niche through malignant JAK-signaling. IL interleukin, FN Fibronectin, HSPC hematopoietic stem and progenitor cell, MSC mesenchymal stem cell, SEMA Semaphorin, SPP1 Osteopontin, TGF Transforming growth factor.

The aberrant activation of bone marrow mesenchymal stroma cells (MSCs) leading to myofibroblast differentiation, excessive secretion of collagen and consequently an increase in reticular fibers and the clinical manifestation of myelofibrosis in MPN is caused by malignant JAK signaling [145, 240] (Fig. 5). Interestingly, uncontrolled activation of JAK-signaling in the absence of MPN-driver mutations is sufficient to cause this phenomenon in experimental model systems. Even before JAK2-V617F was discovered as a genetic driver of MPN, it had been demonstrated, that forced expression of TPO via retroviral expression or hypomorphic mutants in GATA1 were sufficient to cause fibroblast activation and myelofibrosis in mice [241–243]. Mechanistically, this phenomenon was linked to dysfunction of megakaryocytes causing excessive release of TGF-β which in turn stimulated fibroblasts and caused myelofibrosis. Similarly, oncogenic JAK-signaling downstream of MPN driver mutations fuels TGF-β production and the development of myelofibrosis [244–246]. Of note, the role of TGF-β signaling in MSCs for fibrotic transformation is independent of the malignant perturbation of the hematopoietic niche [244]. Aberrantly activated megakaryocytes, therefore, appear to have a dual function in supporting expansion of the MPN clone and promoting fibrosis [245, 247]. Furthermore, bone marrow resident mast cells and T cells stimulate TGF-β production in megakaryocytes by secreting IL4 and IL13 in MPN harboring a JAK2-V617F or MPL-W515L mutation [248, 249]. Comparable to JAK2-V617F, CALR-del52 can also induce TGF-β production, which was connected to the expansion of regulatory T cells in the bone marrow (BM) niche [250]. Chemokine release, particularly CXCL4, from HSPCs appears to be a critical mediator in the fibrosis-promoting microenvironment [240, 251–253]. Of note, targeting IL1β release from HSPCs decreases megakaryopoesis and reduces bone marrow fibrosis [254, 255].

Interestingly, co-mutations also appear to impact the likelihood of myelofibrosis development. A recent study demonstrated, that SRSF2 mutations can impair JAK2-V617F signaling by missplicing of JAK2 leading to reduced TGF-β levels and attenuation of myelofibrosis [256]. The interaction between stroma and hematopoietic cells is also a critical determinant of cell survival and protects JAK2 mutant cells from JAK-inhibitor-induced cell death [257]. Recent publications provide the first evidence that non-malignant “bystander” cells can also contribute to the secretion of pro-inflammatory cytokines, in addition to the JAK2-mutated clone [258]. Cytokine profiles of *JAK2*-mutated cells partially overlap with those of non-malignant “bystanders” or those produced by cells carrying other driver mutations. Secretion of pro-inflammatory cytokines and chemokines causes tissue remodeling in the bone marrow, specifically the development of fibrosis and osteosclerosis [259]. Moreover, the inflammatory milieu may by itself promote myeloproliferation and clonal evolution [260]. Therefore, specific targeting of inflammatory signaling may be a therapeutic opportunity to improve clinical responses in MPN [261]. Of note, targeting the malignant clone as well as bystander inflammation is an inherent feature, particularly of non-selective JAK inhibitors like ruxolitinib. Selective targeting of other inflammatory molecules has been explored mostly in preclinical trials [261] but has not yet led to approval of such a strategy for the treatment of MPN patients. Etanercept, a TNFR2 fusion protein, is used clinically as an anti-inflammatory agent and was tested in an MF trial, improving symptoms similar to ruxolitinib [262]. Antifibrotic treatments like galunisertib, an ALK5 inhibitor, target TGFβ but require combination with agents such as ruxolitinib to address myeloproliferation and clonal burden in MPN mouse models [263].

### Clonal hematopoiesis and end-organ damage

Seminal studies have demonstrated that individuals with clonal hematopoiesis (CHIP) had an increased risk of coronary heart disease and ischemic stroke [4, 264, 265]. Specifically, mutations in genes involved in the JAK-STAT signaling pathway, such as JAK2-V617F, were highlighted as having a particularly strong correlation with adverse cardiovascular outcomes. Moreover, aberrant signaling via non-mutated JAKs downstream of the IL1B/IL6 signaling axis has been identified as the key driver of vascular disease associated with more frequent drivers of CHIP like mutant TET2 and IL6 blockade allowing mitigation of atherosclerosis [266, 267]. Furthermore, elevated levels of pro-inflammatory cytokines, driven by JAK2 mutant clones, contribute to endothelial dysfunction, atherosclerosis, and plaque formation. Emerging evidence suggests that JAK mutations may also play a role in the development of cardiac arrhythmias. In a large cohort study (*n* = 410, 702 participants), the presence of CHIP was associated with a higher incidence of several types of arrhythmias, supraventricular, ventricular, and bradyarrhythmia. The association held true in multivariate analysis, independent of the presence of coronary artery disease or heart failure. The authors carefully conclude that CHIP may constitute a novel risk factor for incident arrhythmias. The mutations are thought to disturb electrical activity, most likely by promoting fibrosis, potentially leading to conditions such as atrial fibrillation [268]. CHIP also increases the risk of acute kidney injury (AKI) as investigated recently across three population-based epidemiology cohorts [269]. The findings revealed that CHIP is associated with a higher risk of incident AKI, particularly in patients requiring dialysis and in those with somatic mutations in JAK2. Mendelian randomization analyzes supported a causal role for CHIP in promoting AKI. In mouse models JAK2-mediated CHIP was associated with more severe AKI, a greater renal pro-inflammatory macrophage infiltration, and increased post-AKI kidney fibrosis. Therefore, JAK2-CHIP contributes to impaired kidney function recovery following AKI through an aberrant inflammatory response mediated by renal macrophages. Furthermore, the presence of a JAK2-V617F mutation is also associated with chronic kidney injury [270, 271]. The impact of JAK2-signaling and the role of JAK2-clonal hematopoiesis in chronic liver disease, on liver health and particularly its role in promoting liver inflammation and injury has been investigated in detail [272]. Individuals with CHIP had a higher risk of developing chronic liver disease. Interestingly, this risk was particularly dependent on the type of CHIP mutation detected. While carriers of a DNMT3A mutation only showed a moderate, insignificant increase in the risk for chronic liver disease (OR: 1.37, *p* = 0.45), individuals with a TET2 mutation showed substantial increase in liver disease risk (OR: 5.35, *p* < 0.001). Moreover, individuals with an activating JAK2 mutation showed the highest risk for the development of chronic liver disease (OR: 17.65, *p* < 0.001) highlighting the genotype-specific risk of these patients and the need for longitudinal monitoring of liver function and a consequent reduction of additional lifestyle-related risk factors in this subset of patients [272]. Mice with clonal mutations, particularly in the Jak2 gene, exhibited increased liver inflammation and fibrosis following liver injury. The presence of these mutations led to heightened activation of the NLRP3 inflammasome in liver macrophages, resulting in elevated levels of pro-inflammatory cytokines and chemokines. Therefore, this study highlights JAK2-mediated CHIP as a potentially modifiable risk factor for chronic liver disease and suggests that targeting JAK-signaling or its downstream effector NLRP3 could provide a strategy to mitigate this risk [272]. NLRP3 activation and consecutive IL-1β release were shown to drive myeloproliferation in mice [273].

### Unique functions of JAKs in immune cells

MPN patients treated with the JAK1/2 inhibitor ruxolitinib incurred a higher incidence of viral infections in early clinical studies [274, 275]. JAK1/2 inhibition decreased the number of CD3+ T-cells and reduced cytokine production. Th1 cells and regulatory T-cells (Treg) appear to be the most significantly impacted [276–278]. However, JAK1/2 inhibitors also impair CD8+ T-cell effector activity [279]. JAK inhibition also impairs dendritic cell function [280], B-cell differentiation, and antibody production [281, 282]. Because the inhibition of cellular processes by JAK-inhibition is partial and unique to specific cell types, JAK inhibition is comparatively well tolerated by patients [140, 142, 275, 283] Clinical trials noted a low incidence of side effects related to infections. Consequently, rather than being a traditional immunosuppressive medication, JAK-inhibitor should be viewed as an immunomodulatory therapy. As noted above, pharmacologic perturbation of JAK-signaling is also successfully employed in the treatment of acute and chronic GvHD following allogeneic stem cell transplantation [153]. Ruxolitinib increased survival and reduced the generation of proinflammatory cytokines and Th1 and Th17 polarization in a mouse model of GvHD. In a pilot study, ruxolitinib effectively reduced clinical symptoms and cytokine production in individuals with steroid-resistant GvHD who were extensively pre-treated[150]. Ruxolitinib is EMA-approved for the treatment of steroid-refractory acute and chronic GvHD in adult and pediatric patients aged 12 years and older [284].

Paradoxically and importantly, the graft-versus-leukemia effect, which is necessary for therapeutic success of allogeneic stem cell transplantation, was not impacted by ruxolitinib treatment [285]. This observation supports the hypothesis that JAK inhibition has selective immunomodulatory activities. It is still unclear to what extent distinct JAKs perform redundant and non-redundant tasks in immune cells. When using more selective JAK2-inhibitors (such as fedratinib) selective JAK2-suppression had no such effect. In vitro, treatment of healthy donor T-cells with either ruxolitinib or momelotinib, both JAK1/2 inhibitors, led to inhibition of proliferation, global activation (CD69), and paradoxically, compensatory STAT1 phosphorylation in CD4+ and CD8+ T-cells [286]. These effects were validated using RNA interference (RNAi). In these assays, the only way to suppress global T-cell activity in vitro was to deplete JAK1. Consistent with this observation, JAK1 but not JAK2 was required for global T-cell effector activities in a mouse model of GvHD [286]. These results emphasize the significance of JAK-selectivity in relation to the context or the underlying pathophysiology. Clinical trials are presently being conducted to examine JAK1, JAK3, and TYK2 selective inhibitors for several inflammatory disorders [287–298]. Oncogenic JAK2-V617F mutations in MPNs also promote immune evasion by upregulating PD-L1 through STAT3/STAT5 activation [299]. Inhibition of JAK2 reduces PD-L1 expression while blocking PD-1 improves survival in MPN models by restoring T cell function. PD-L1-mediated immune escape primarily affects monocytes, megakaryocytes, and platelets, impairing T cell metabolism and cell cycle progression. These findings highlight PD-1 inhibition as a potential therapeutic strategy in JAK2-mutant MPNs [299]. With the availability of JAK2-selective inhibitors that do not restrict global T-cell functions, immune checkpoint inhibitors might enable effective immunotherapy in MPN patients in the future. A clinical trial probing this combination is currently underway [300].

## Outlook: challenges and perspectives regarding malignant JAK-signaling

Malignant JAK-signaling presents several clinical challenges, including clonal evolution of JAK-mutated cells, disease persistence, and progression, and an inflammatory phenotype that significantly contributes to morbidity and progression risk.

### Evolution of JAK-mutated clones

The JAK2V617F mutation, a primary driver in MPNs, promotes increased hematopoietic stem cell (HSC) division and DNA damage but with a relatively low mutation rate, about one mutation per 66 patient-years. This indicates that hypermutability is not the primary driver of MPN progression [140, 301]. Studies using lineage tracing and phylogenetic reconstruction show that the JAK2 mutation often emerges decades before clinically overt MPN is diagnosed, sometimes even in utero [302]. The long lag period implies that acquisition of the mutation alone doesn’t necessarily lead to disease but may require additional genetic or environmental events. Individuals who harbor only the JAK2-V617F mutation, frequently do not progress to overt MPN but may achieve long-term clonal stability or even regression, suggesting a degree of cellular adaptation or immune regulation. However, in individuals carrying additional mutations (e.g., TET2, DNMT3A), the risk for malignant transformation increases significantly. These observations emphasize that the sequence in which mutations are acquired as well as the type of mutations incurred influence disease trajectory and phenotypic manifestation. For example, JAK2 as a primary mutation often leads to polycythemia vera (PV), whereas, as a secondary event, it commonly results in essential thrombocythemia (ET) [303].

### Disease persistence and progression of JAK-mutated MPN

Disease persistence in MPNs is driven by dynamic clonal evolution where JAK2-mutant clones incur additional somatic mutations, typically in genes associated with DNA repair, epigenetic regulation, and signaling pathways [304, 305]. Notably, the mutation hierarchy changes over time, influenced by therapeutic pressures. For example, ASXL1 mutations which are considered high-risk somatic mutations are selected for under ruxolitinib therapy [306]. Even with therapies like interferons, which effectively reduce clonal burden, and JAK inhibitors, which manage symptoms and splenomegaly, complete disease eradication remains rare [301, 307]. Almost ubiquitous MPN disease persistence emphasizes the need for therapies targeting abnormalities at the stem cell level, to fully prevent disease progression and malignant transformation.

### The inflammatory phenotype in JAK-mutated MPN

Chronic inflammation, a hallmark of JAK-driven MPN, significantly influences disease progression and symptom burden. Elevated pro-inflammatory cytokines such as TNF-α and IL-8 are found in JAK2-mutated clones and correlate with higher disease burden and adverse outcomes [301, 308]. Persistent JAK-STAT pathway activation in malignant and non-malignant cells promotes a pro-inflammatory environment that exacerbates MPN pathology and can further stimulate clonal growth, fibrosis, and vascular events [309, 310]. This inflammatory milieu is linked to the progression of MPN to myelofibrosis or even acute leukemia, necessitating anti-inflammatory interventions.

### Therapeutic perspectives

Among current therapeutic options, the JAK inhibitors ruxolitinib, fedratinib, momelotinib, and pacritinib offer symptom relief and reduce spleen size but often fail to halt clonal evolution comprehensively [309, 311]. Additionally, apoptosis-inducing agents like navitoclax and navtemadlin, epigenetic modulators like pelabresib and bomedemstat, and telomerase inhibitors like imetelstat are emerging as promising combination partners with JAK inhibitors [312]. These agents target the malignant clone at various molecular levels, addressing both the survival advantage of MPN cells and the associated inflammatory signaling. Effective integration of these novel therapies with JAK inhibition could offer better control over disease progression and QoL improvement [313, 314]. In conclusion, addressing the complexities of JAK-driven MPN will require an integrated approach that combines targeted molecular therapies with anti-inflammatory treatments. Future therapies should focus on modifying the inflammatory microenvironment, slowing clonal expansion, and intervening at the stem cell level to prevent disease evolution and progression.
